# The mitochondrial-derived peptide MOTS-c improves bioenergetics in aging-associated granulosa cells and oocytes

**DOI:** 10.1530/RAF-26-0161

**Published:** 2026-10-27

**Authors:** Kuan-Hao Tsui, Shih-Hsuan Cheng, Li-Te Lin, Pei-Hsuan Lin, Boyang Wang, Zhi-Hong Wen, Chia-Jung Li

**Affiliations:** ^1^ Department of Obstetrics and Gynecology, Kaohsiung Veterans General Hospital, Kaohsiung, Taiwan; ^2^ Institute of Biopharmaceutical Sciences, National Sun Yat-sen University, Kaohsiung, Taiwan; ^3^ Department of Obstetrics and Gynecology, National Yang-Ming University School of Medicine, Taipei, Taiwan; ^4^ National Museum of Marine Biology & Aquarium, Pingtung, Taiwan; ^5^ School of Medicine, College of Medicine, National Sun Yat-sen University, Kaohsiung, Taiwan; ^6^ Center of General Education, Cheng Shiu University, Kaohsiung, Taiwan; ^7^ Sundial Initiative, Palo Alto, California, USA; ^8^ Department of Marine Biotechnology and Resources, National Sun Yat-sen University, Kaohsiung, Taiwan

**Keywords:** MOTS-c, mitochondrial-derived peptide, granulosa cell metabolism, oocyte bioenergetics, mitochondrial quality control, reproductive senescence

## Abstract

**Abstract:**

Ovarian aging compromises granulosa cell function, mitochondrial homeostasis, metabolism, and oocyte quality. We investigated whether the mitochondrial-derived peptide MOTS-c mitigates these defects using an aging-associated HGL5 granulosa cell model, *ex vivo* oocytes from young and aged mice, and public granulosa cell transcriptomic datasets. In HGL5 cells, MOTS-c increased mitochondrial mass and ATP-associated fluorescence without increasing cellular or mitochondrial reactive oxygen species, while mass-normalized membrane potential remained unchanged. Seahorse analysis showed increased ATP-linked and maximal respiration and spare respiratory capacity, reduced proton leak, and unchanged basal respiration. MOTS-c also remodeled the expression of enzymes involved in glycolysis, lactate–pyruvate handling, and pyruvate–TCA metabolism. Although fission markers were largely unchanged, MOTS-c increased the levels of proteins associated with antioxidant defense, mitochondrial transport, cell junctions, adhesion signaling, and granulosa cell function, including NRF2, phosphorylated AIFM1, RHOT1, KIF5B, ZO-1, E-cadherin, phosphorylated FAK, and AMH. Aged oocytes exhibited reduced polar body extrusion, increased DCFDA fluorescence, and decreased CMXRos intensity. *Ex vivo* MOTS-c treatment increased post-culture first polar body positivity and mitochondria-associated fluorescence while reducing oxidative stress-associated fluorescence, although outcomes remained below young-reference levels. Reanalysis of human folliculogenesis and nonhuman primate ovarian aging datasets further revealed a young-enriched trend in the MOTS-c-associated metabolic program. These findings suggest that MOTS-c improves metabolic, mitochondrial, and structural features of aging-associated granulosa cells and enhances maturation-associated outcomes in aged oocytes, supporting further investigation of MOTS-c-based strategies for reproductive aging.

**Lay summary:**

As women age, the quality of their eggs declines, which is a major contributor to age-related infertility. Eggs are supported by neighboring ‘helper’ cells called granulosa cells, which act like a support crew that supplies the egg with the energy it needs to mature. These helper cells contain mitochondria, tiny energy-generating structures often called the cell’s ‘powerhouses’, and with age these powerhouses become less efficient, leaving eggs with less support than they need. This study tested whether MOTS-c, a small molecule naturally produced by mitochondria, could restore the function of these aging helper cells. In laboratory experiments using aged human granulosa cells and eggs from aged mice, MOTS-c boosted energy production and helped maintain cell structure. It was also associated with improved egg maturation compared with that in untreated aged eggs, although treated eggs did not fully reach the maturation levels seen in young mice. These findings are from laboratory and animal experiments, not from women, and this study did not test whether MOTS-c affects fertilization, embryo development, or live birth. Further research is needed before any conclusions can be drawn about its relevance to human fertility.

## Introduction

Ovarian aging is a major factor in the age-related decline in female reproductive potential and is marked by a progressive loss of follicular reserve and reduced oocyte quality and competence (Li *et al.* 2021, Hirano *et al.* 2025). Within the follicular unit, oocyte development depends on a regulated somatic microenvironment, with granulosa and cumulus cells providing metabolic and structural support (Morimoto *et al.* 2024, Wang 2025). Because mammalian oocytes have a limited capacity for glucose uptake and glycolysis, they rely on surrounding follicular somatic cells to metabolize glucose and supply energy substrates, including pyruvate and lactate (Zhang *et al.* 2022, Zhang *et al.* 2025). This metabolic coupling, together with bidirectional somatic–germline communication, is required for follicular homeostasis, oocyte developmental competence, and meiotic progression (Johnson *et al.* 2007).

Beyond chronological aging, metabolic disorders such as obesity, lipotoxicity, and insulin resistance are increasingly recognized as contributors to an accelerated, aging-like decline in oocyte and granulosa cell function, in part through overlapping mechanisms of mitochondrial dysfunction and metabolic stress within the ovarian microenvironment (Morimoto *et al.* 2024). Because MOTS-c is a mitochondrial-derived peptide with systemic roles in metabolic homeostasis and insulin sensitivity (Lee *et al.* 2015), understanding how metabolic stress affects the follicular microenvironment provides additional biological rationale for evaluating this peptide in models of reproductive aging.

Evidence indicates that mitochondrial dysfunction in granulosa cells contributes to ovarian aging (Ju *et al.* 2024). Beyond steady-state ATP production, the ability of granulosa cells to maintain mitochondrial respiratory reserve and adapt to energetic stress may be needed to support oocyte maturation and developmental competence. Age-related impairment of oxidative phosphorylation, altered metabolic flexibility, and excess accumulation of reactive oxygen species may disrupt the metabolic support provided by granulosa cells to the oocyte (Liu *et al.* 2017). This energetic imbalance is associated with reduced oocyte quality and remains a barrier to reproductive aging interventions (Lewis *et al.* 2020). Although antioxidant-based approaches have been tested, their clinical effects remain limited and variable, in part because of differences in study design and the lack of well-defined intracellular metabolic targets within the aging follicular microenvironment (Camerano Spelta Rapini *et al.* 2026).

Mitochondrial-derived peptides (MDPs) have been identified as bioactive mediators of cellular stress adaptation and aging-related metabolic regulation (Mohtashami *et al.* 2022). MOTS-c is a 16-amino-acid peptide encoded within a short open reading frame in the mitochondrial 12S rRNA region and has been linked to mitonuclear retrograde signaling (Lee *et al.* 2015). Under metabolic stress, MOTS-c can translocate to the nucleus and regulate nuclear gene expression through AMPK-dependent stress-responsive pathways, including NRF2-associated transcriptional programs (Kim *et al.* 2018*a*). Studies in male reproductive models suggest that MOTS-c may contribute to central reproductive regulation, as central MOTS-c infusion increased LH, FSH, and testosterone release in rats (Ozturk Öztürk *et al.* 2024). MOTS-c has also been reported to reduce prepubertal cyclophosphamide-induced reproductive injury in male mice (Wang *et al.* 2024). Whether MOTS-c can directly improve aging-associated granulosa cell dysfunction and support the somatic environment required for oocyte maturation remains unclear.

In the ovarian follicle, metabolic support and structural organization are functionally linked. Somatic–oocyte support depends not only on mitochondrial fitness but also on the maintenance of cell–cell adhesion and junction-associated architecture (Morimoto *et al.* 2024, Emery *et al.* 2025). Adhesion- and junction-associated proteins, including E-cadherin and ZO-1, contribute to the intercellular organization required for follicular integrity, whereas mitochondrial trafficking regulators such as RHOT1/Miro1 help coordinate mitochondrial distribution in cells with high local energy demand (Zhang *et al.* 2018*a*,*b*, Fagbadebo *et al.* 2022, Zeng *et al.* 2025). During ovarian aging, disruption of metabolic, mitochondrial, and structural programs may weaken the ability of granulosa cells to sustain oocyte maturation. These observations suggest that recovery of granulosa cell function may require coordinated improvement of a metabolic–structural support axis.

In this study, we investigated whether MOTS-c improves aging-associated granulosa cell dysfunction through the regulation of mitochondrial bioenergetics, metabolic remodeling, and somatic structural support. Using an aging-associated human granulosa cell model, *ex vivo* assays with denuded oocytes from young and aged mice, and public granulosa cell transcriptomic datasets spanning human and nonhuman primate samples, we evaluated the role of MOTS-c in supporting the granulosa cell microenvironment and improving meiotic maturation-associated outcomes during reproductive aging. Because MOTS-c was applied directly to denuded oocytes in the *ex vivo* assays, these two lines of evidence – granulosa cell support function and oocyte-associated outcomes – are considered independent findings rather than a proven mechanistic axis. Our findings suggest that MOTS-c is a candidate regulator of granulosa cell metabolic flexibility and follicular support, providing a basis for future studies of peptide-based strategies to improve oocyte quality.

## Materials and methods

### Cell culture and MOTS-c treatment

Human granulosa-like HGL5 cells were obtained from Applied Biological Materials Inc. and maintained in DMEM/F12 medium containing 10% fetal bovine serum, 1% penicillin–streptomycin, 2% Ultroser G, and 1% ITS Plus. Cells were cultured at 37°C in a humidified incubator with 5% CO_2_. Late-passage HGL5 cells, defined as cells at passages greater than 50, were used to model aging-associated granulosa cell dysfunction. These cells were obtained by subculturing early-passage (<P10) cells every 2 days for approximately 3 months. This late-passage HGL5 model has been previously validated by our group, which reported senescence-associated mitochondrial and functional changes relative to early-passage cells (Li *et al.* 2022). Consistent with this validation, late-passage (aged) HGL5 cells used in the present study showed increased SA-β-galactosidase activity and increased p16 and p27 protein expression relative to early-passage (young) HGL5 cells (Supplementary Fig. S1A and B (see the section on Supplementary materials given at the end of the article)). MOTS-c (Targetmol, Catalog No. TP2312; purity 99.93%; Lot 146627) was prepared as a stock solution in double-distilled water and diluted into complete culture medium immediately before use. A dose–response analysis of ATP-associated fluorescence across a range of MOTS-c concentrations (0–100 μM, 24 h) showed a near-saturating response at 50 μM (Supplementary Fig. S1C), supporting the selection of this concentration for HGL5 cell and oocyte experiments. Unless otherwise stated, cells were exposed to 50 μM MOTS-c for 24 h, while control cells received the same volume of vehicle.

### Flow cytometric assessment of mitochondrial status and redox-related signals

To evaluate mitochondria-associated functional readouts, control and MOTS-c-treated HGL5 cells were collected after treatment and washed with PBS before fluorescent staining. Mitochondrial content was assessed using MitoTracker Green, mitochondrial membrane potential-associated signal was measured using TMRE, intracellular ATP-associated fluorescence was detected using BioTracker ATP-Red Live Cell Dye, total cellular ROS was evaluated using DCFDA, and mitochondrial superoxide was measured using MitoSOX Red. Because an increase in total mitochondrial mass can independently elevate the absolute intensity of potentiometric dyes, TMRE fluorescence was also normalized to MitoTracker Green fluorescence on a per-sample basis to derive a mass-corrected index of membrane potential. Staining was performed at 37°C in the dark according to the recommended incubation conditions for each probe. After staining, cells were washed to remove excess dye, resuspended in PBS, and analyzed immediately by flow cytometry.

### Seahorse extracellular flux analysis

Mitochondrial respiratory activity was examined using a Seahorse XF HS Mini extracellular flux analyzer. HGL5 cells were seeded into Seahorse assay plates at 2,000 cells per well and treated with vehicle or MOTS-c before analysis. Oxygen consumption rate was recorded during the mitochondrial stress test. Oligomycin (1 μM), FCCP (1 μM), and antimycin A/rotenone (0.5 μM) were sequentially injected to determine basal respiration, ATP-linked respiration, maximal respiration, spare respiratory capacity, proton leak, and nonmitochondrial respiration. OCR values were normalized to total cellular protein content per well, determined by BCA assay at the conclusion of the assay. Experiments were independently repeated 3–5 times, with three technical replicate wells per condition in each experiment; no wells were excluded from analysis.

### Western blot analysis

Western blotting was performed as previously described (Li *et al.* 2015). Briefly, total protein was extracted from control and MOTS-c-treated HGL5 cells, separated by SDS-PAGE, and transferred onto membranes for immunoblotting. The following primary antibodies were used: ZO-1 (#5406; Cell Signaling Technology, USA); RHOT1 (GTX3283), Paxillin (GTX633769), phospho-FAK Tyr397 (GTX129840), FAK (GTX82780), α-tubulin (GTX628802), β-actin (GTX629630), and AMH (GTX129593) from GeneTex, Taiwan; phospho-Drp1 Ser637 (ab193216) and Drp1 (ab56788) from Abcam, UK; E-cadherin (20874-1-AP), MID49 (28718-1-AP), MID51 (20164-1-AP), and NRF2 (16396-1-AP) from Proteintech, USA; phospho-Drp1 Ser616 (AP0849), KIF5B (A15284), HK2 (A0994), ENO1 (A1033), PKM (A0268), LDHA (A1146), LDHB (A7625), PDHA1 (A1895), CS (A5713), IDH1 (A2169), ACO1 (A7867), and FH (A5688) from ABClonal, USA; AIFM1 (E-AB-10123) from Elabscience, USA; and phospho-AIFM1 Ser116 (AP5501) from ECM BD Biosciences (USA). β-actin or α-tubulin was used as the loading control, as appropriate; loading control band intensities did not differ appreciably between the control and MOTS-c-treated groups. Phospho-AIFM1 and phospho-FAK Tyr397 were normalized to total AIFM1 and total FAK, respectively, and phospho-DRP1 (Ser637 and Ser616) was normalized to total DRP1, whereas other protein levels were normalized to β-actin or α-tubulin. Band intensities were quantified by densitometry and expressed relative to control values.

### Animal experiments and *ex vivo* oocyte culture

Female C57BL/6J mice were obtained from the National Laboratory Animal Center and housed under controlled temperature and light-cycle conditions with free access to food and water. All animal procedures were approved by the Institutional Animal Care and Use Committee of Kaohsiung Veterans General Hospital (IACUC-2508-2807-25002). Young (<12 weeks) and aged (>34 weeks) female mice were used for oocyte-related experiments to allow comparisons across reproductive ages. Superovulation was induced in both age groups by intraperitoneal injection of 5 IU equine chorionic gonadotropin, followed 48 h later by 5 IU human chorionic gonadotropin. Cumulus–oocyte complexes were collected approximately 14–16 h after hCG administration. Cumulus cells were removed using hyaluronidase or ICSI Cumulase, and denuded oocytes were randomly assigned to vehicle or MOTS-c (50 μM, matching the concentration used in HGL5 cell experiments) treatment for 24 h of *ex vivo* culture at 37°C under 5% CO_2_. Young oocytes were cultured under identical conditions without MOTS-c as a young reference group. The 24-h *ex vivo* culture period was designed to assess whether aged oocytes with delayed meiotic progression could complete first polar body (PB1) extrusion in the presence or absence of MOTS-c. Oocytes retaining a compact granulosa cell layer that resisted enzymatic denudation were recorded as a distinct morphological finding. This appearance alone was not considered sufficient to establish germinal vesicle-stage arrest or an oocyte maturation stage. The first polar body extrusion rate was scored by a research assistant blinded to the treatment group.

### Fluorescent staining of mouse oocytes

After *ex vivo* culture, denuded oocytes were subjected to fluorescence-based assessment of redox- and mitochondria-associated signals. Intracellular ROS was detected using CM-H_2_DCFDA, and mitochondria-associated fluorescence was evaluated using CMXRos, a cationic dye whose mitochondrial accumulation is membrane potential-dependent according to the manufacturer’s specifications. Fluorescence intensity was therefore interpreted as a relative mitochondrial activity/membrane potential-associated index rather than a ratiometric, absolute measurement. Oocytes were incubated with the indicated probes at 37°C in the dark, washed after staining, and imaged using fluorescence microscopy under identical exposure settings across groups. Mean fluorescence intensity was calculated after subtraction of the local background signal and expressed relative to oocyte area. In the small number of oocytes with residual granulosa cells remaining after attempted denudation, fluorescence signals attributable to these cells were excluded from the quantification of oocyte-associated DCFDA and CMXRos intensity.

### Public transcriptomic validation

Public transcriptomic datasets were analyzed to examine whether the MOTS-c-associated granulosa cell program identified experimentally was represented in independent transcriptomic data. GSE130664, a single-cell RNA-sequencing dataset from young and aged cynomolgus monkey (Macaca fascicularis) ovaries, was used for age-associated single-cell analysis of granulosa cells (Wang *et al.* 2020), and GSE107746, an RNA-sequencing dataset of human granulosa cells sampled across five folliculogenesis stages, was used as an independent human folliculogenesis-stage reference (Zhang *et al.* 2018*a*,*b*). For GSE130664, UMI counts were processed using Seurat. Cell barcodes were matched to metadata, gene symbols were standardized, and low-quality cells were removed using thresholds of nFeature_RNA ≥ 200, nFeature_RNA ≤ 6,000, and percent.mt ≤ 15. Data were normalized, scaled, clustered, and visualized by UMAP using dimensions 1–20 and a clustering resolution of 0.4. Donor groups were assigned according to the raw_data prefix, with YF defined as young and OF as aged. Granulosa-enriched cells were selected from cluster 2 based on AMH, INHA, INHBA, AMHR2, CYP19A1, and FSHR expression. MOTS-c-associated metabolic, mitochondrial protection/trafficking, junction/support, and OXPHOS modules were scored using a deterministic z-mean approach, defined as the mean of gene-wise z-scored expression values across all genes in each module. The metabolic program module comprised HK2, ENO1, PKM, LDHC, LDHB, PDHA1, CS, ACO1, and IDH1; the mitochondrial protection/trafficking module comprised NFE2L2, AIFM1, RHOT1, and KIF5B; and the junction/support module comprised TJP1 and CDH1. The OXPHOS module used the MSigDB HALLMARK_OXIDATIVE_PHOSPHORYLATION gene set. Donor-level median scores were used for young-versus-aged comparisons to avoid cell-level pseudoreplication, and statistical comparisons were performed using Wilcoxon rank-sum tests. For GSE107746, follicular stages were inferred from sample names and analyzed as an ordered stage reference. Stage-level module variation was evaluated using Kruskal–Wallis tests and Spearman trend analysis.

### Statistical analysis

Quantitative data are presented as mean ± standard deviation unless otherwise indicated. Image-based fluorescence intensity was quantified using ImageJ or ZEN lite software under consistent acquisition and analysis settings. Statistical comparisons between two experimental groups were performed using an unpaired two-tailed Student’s *t*-test, and comparisons among the young, aged, and aged + MOTS-c oocyte groups were performed using one-way ANOVA with Tukey’s post hoc test. Formal normality testing was not performed for these small-sample-size comparisons; parametric tests were applied as a standard convention for these assay types, and this is noted as a limitation. For public transcriptomic analyses, donor-level comparisons were performed using Wilcoxon rank-sum tests, and multiple comparisons were adjusted using the Benjamini–Hochberg method. Folliculogenesis-stage analyses were evaluated using Kruskal–Wallis tests and Spearman trend analysis. Statistical analyses were conducted using GraphPad Prism and R. A *P* value below 0.05 was considered statistically significant.

## Results

### MOTS-c enhances mitochondrial energetic status without increasing oxidative stress in aged granulosa cells

To determine whether MOTS-c improves mitochondrial functional status in aged granulosa cells, we first assessed mitochondria-associated fluorescence signals by flow cytometry. MOTS-c treatment markedly increased MitoTracker Green fluorescence, consistent with an increase in mitochondrial mass/biogenesis (Fig. 1A). ATP-associated fluorescence was also significantly elevated in MOTS-c-treated cells compared with control cells (Fig. 1C). Because an increase in mitochondrial mass can independently elevate the absolute potentiometric dye signal, TMRE fluorescence was normalized to MitoTracker Green fluorescence on a per-sample basis to derive a mass-corrected index of membrane potential; this index was not significantly different between control and MOTS-c-treated cells (Fig. 1B), indicating that the increase in mitochondrial mass shown in (A) was not accompanied by a proportional, mass-independent increase in membrane potential. In contrast, total cellular ROS, assessed by DCFDA, and mitochondrial superoxide, assessed by MitoSOX, were not significantly increased after MOTS-c treatment (Fig. 1D and E). These results indicate that MOTS-c increases mitochondrial mass and intracellular ATP-associated signal without inducing detectable oxidative stress in aged granulosa cells.

**Figure 1:**
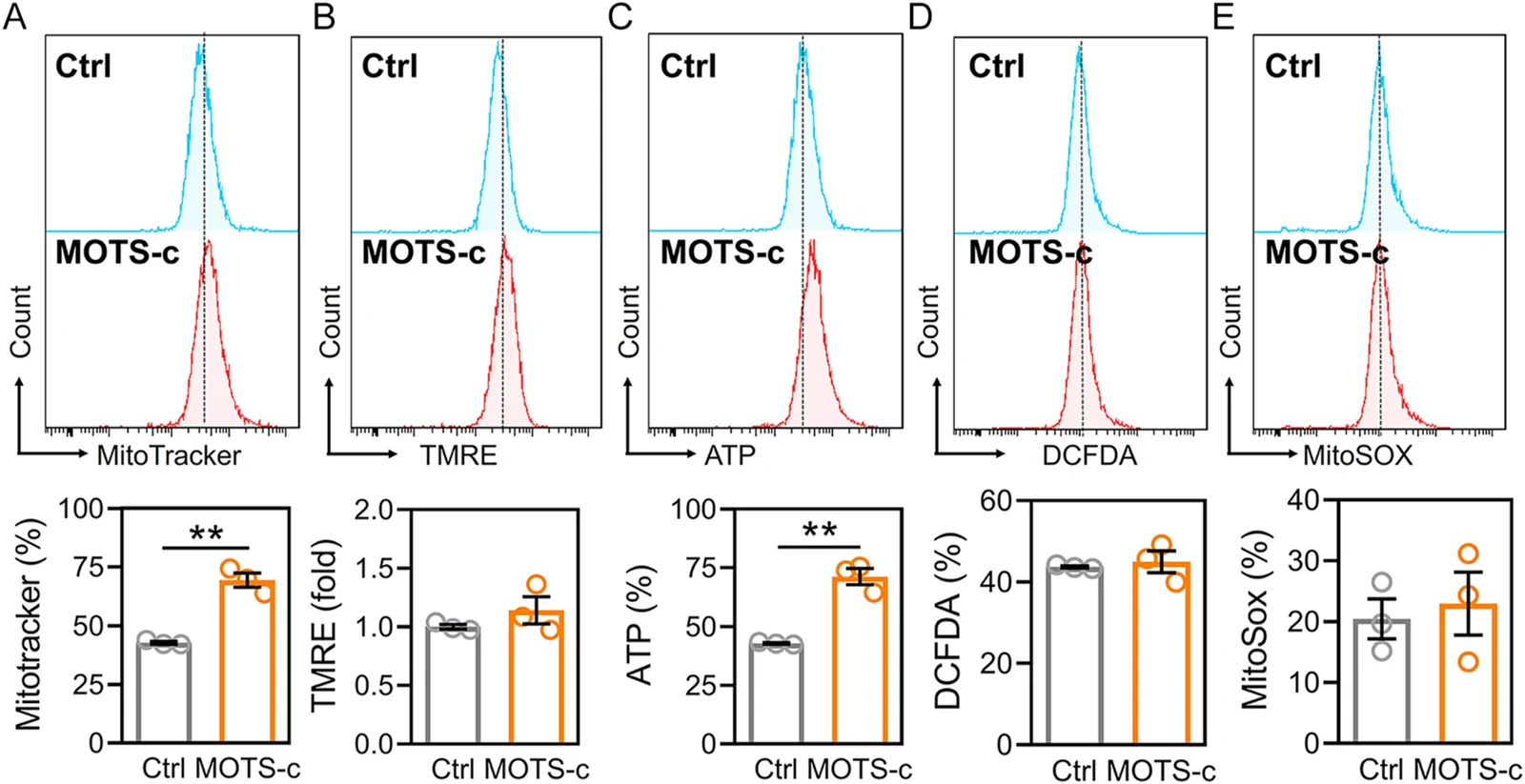
MOTS-c increases mitochondrial mass and ATP-associated signal without increasing oxidative stress in aged granulosa cells. (A) Representative histogram and quantification of MitoTracker Green fluorescence showing increased mitochondria-associated signal (mitochondrial mass/biogenesis) after MOTS-c treatment. (B) Representative TMRE histogram and quantification of TMRE fluorescence normalized to MitoTracker Green fluorescence (a mass-corrected index of mitochondrial membrane potential), showing no significant difference between control and MOTS-c-treated cells, indicating that the increase in mitochondrial mass shown in (A) was not accompanied by a proportional, mass-independent increase in membrane potential. (C) Representative ATP-associated fluorescence histogram and quantification showing increased intracellular ATP-associated signal after MOTS-c treatment. (D) Representative DCFDA histogram and quantification showing no significant increase in total intracellular ROS after MOTS-c treatment. (E) Representative MitoSOX histogram and quantification showing no significant increase in mitochondrial superoxide. **P < 0.01.

### MOTS-c improves mitochondrial respiratory performance and reserve capacity

We next examined mitochondrial respiration using Seahorse extracellular flux analysis. The overall oxygen consumption rate profile showed that MOTS-c-treated cells displayed a stronger respiratory response after FCCP stimulation than control cells (Fig. 2A). Quantitative analysis showed that basal respiration was not significantly altered by MOTS-c treatment (Fig. 2B), indicating that MOTS-c did not simply increase steady-state oxygen consumption. Instead, MOTS-c significantly increased ATP-linked respiration, maximal respiration, and spare respiratory capacity (Fig. 2C, D, E). Proton leak was significantly reduced, whereas nonmitochondrial oxygen consumption was not significantly changed (Fig. 2F and G). These findings suggest that MOTS-c improves mitochondrial respiratory efficiency and reserve capacity, supporting a more competent bioenergetic state rather than a nonspecific increase in basal respiration.

**Figure 2:**
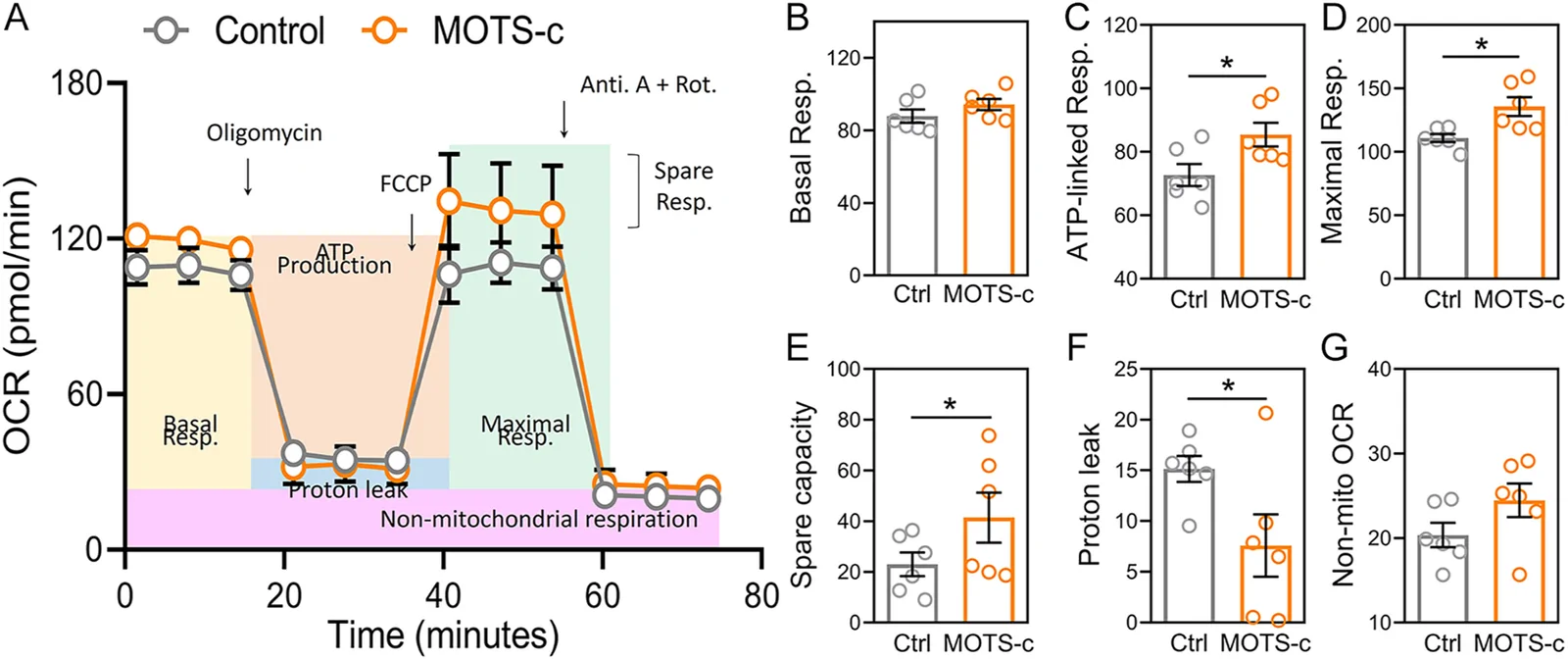
MOTS-c improves mitochondrial respiratory performance and reserve capacity. (A) Representative OCR profile following sequential injection of oligomycin, FCCP, and antimycin A/rotenone. (B) Quantification of basal respiration showing no significant change after MOTS-c treatment. (C) MOTS-c significantly increased ATP-linked respiration. (D) MOTS-c significantly increased maximal respiration. (E) MOTS-c significantly enhanced spare respiratory capacity. (F) MOTS-c significantly reduced proton leak. (G) Nonmitochondrial OCR was not significantly altered. *P < 0.05.

### The granulosa metabolic program declines with aging and is reinforced by MOTS-c toward pyruvate–TCA utilization

We first asked whether the metabolic machinery examined here is physiologically engaged during follicular development. Across inferred folliculogenesis stages in human granulosa cells, metabolic, mitochondrial protection/trafficking, and junction/support genes showed stage-associated expression, indicating that these programs are actively used throughout normal follicle maturation (Fig. 3A). We next examined whether this program is altered with reproductive aging. At the donor level, the MOTS-c metabolic program score was higher in young than in aged granulosa cells (Fig. 3B), whereas the OXPHOS module showed substantial donor-to-donor overlap between age groups without a consistent young-enriched separation (Fig. 3C). These transcriptomic observations indicate that the metabolic component of the program is the most consistently age-attenuated feature, motivating direct testing of whether MOTS-c can reinforce metabolic enzyme expression in aging-associated granulosa cells. To this end, we examined key enzymes involved in glycolysis, lactate handling, pyruvate entry, and the TCA cycle (Fig. 3D, E, F, G). MOTS-c significantly increased the protein levels of HK2, ENO1, and PKM1, indicating enhanced glycolytic processing (Fig. 3D and E). Among LDH isoforms, MOTS-c decreased LDHA while increasing LDHB, suggesting remodeling of lactate-associated metabolism rather than uniform activation of lactate production (Fig. 3E). MOTS-c also increased PDHA, CS, ACO1, and IDH1, supporting enhanced pyruvate entry and activation of selected TCA-cycle-associated nodes, whereas FH was not significantly altered (Fig. 3F and G). Together, these results suggest that the granulosa metabolic program is engaged during folliculogenesis and attenuated with aging, and that MOTS-c reinforces glycolytic, lactate-associated, and pyruvate–TCA metabolic proteins, potentially contributing to the improved mitochondrial respiratory phenotype observed in Seahorse analysis.

**Figure 3:**
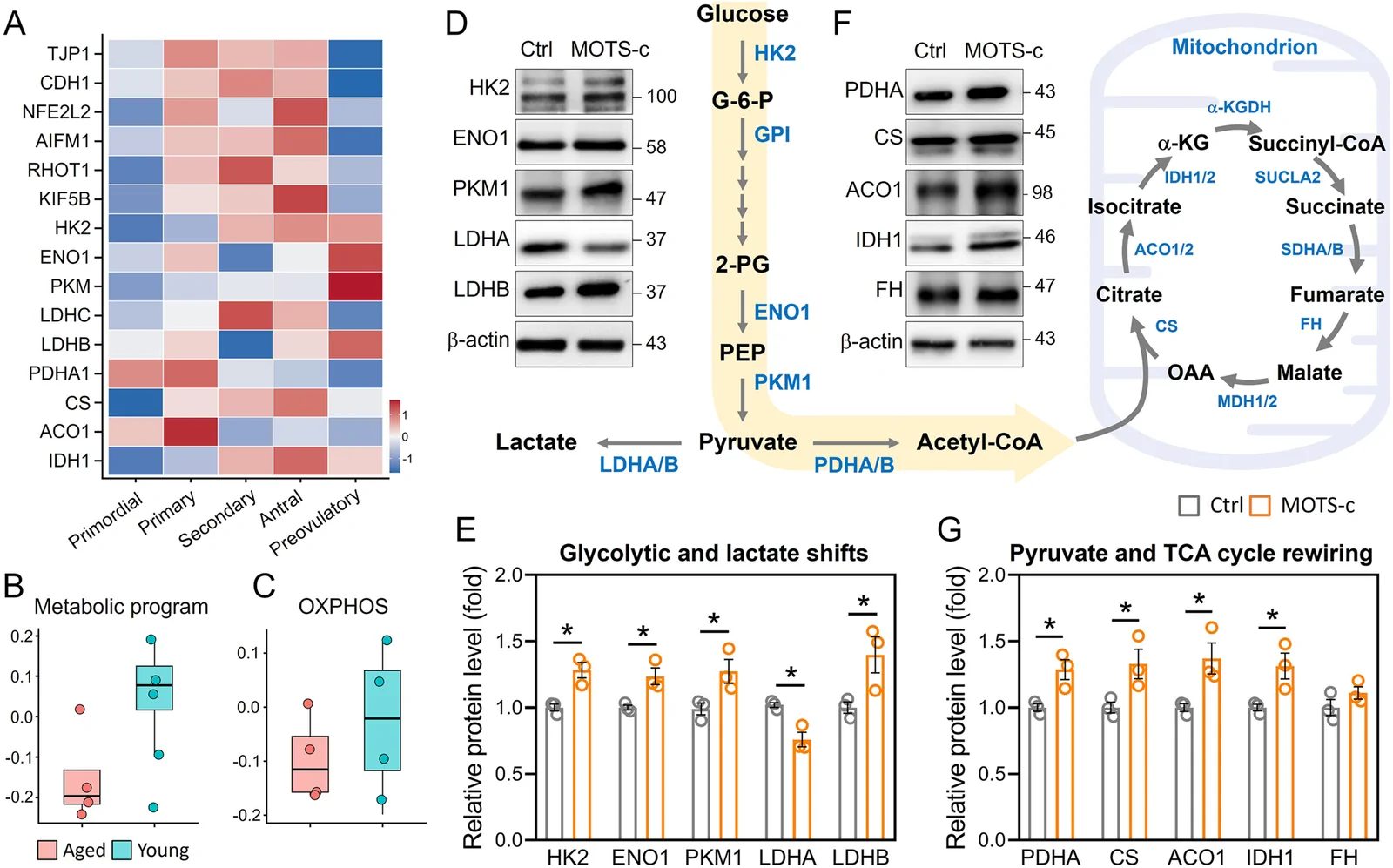
The granulosa metabolic program is engaged during folliculogenesis, attenuated with aging, and reinforced by MOTS-c toward pyruvate–TCA utilization. (A) Heatmap of MOTS-c-associated genes (metabolic, mitochondrial protection/trafficking, and junction/support) across inferred folliculogenesis stages in human granulosa cells (GSE107746), showing stage-associated expression. Values were averaged within each stage and z-scored within each gene. (B) Donor-level comparison of the MOTS-c metabolic program score in aged versus young granulosa cells (GSE130664), showing a young-enriched distribution. (C) Donor-level comparison of the OXPHOS module score, which showed substantial overlap between age groups without a consistent separation. (D) Representative blots of glycolytic and lactate-associated enzymes and (E) corresponding quantification. MOTS-c increased HK2, ENO1, PKM1, and LDHB while reducing LDHA, suggesting enhanced glycolytic processing and altered lactate–pyruvate handling. (F) Representative blots of pyruvate–TCA-associated enzymes and (G) corresponding quantification. MOTS-c increased PDHA, CS, ACO1, and IDH1, whereas FH was not significantly changed, suggesting increased pyruvate–TCA metabolic readiness. The schematic diagrams indicate the analyzed metabolic nodes. β-actin was used as the loading control. *P < 0.05.

### MOTS-c promotes mitochondrial protective trafficking and structural support markers in granulosa cells

We next examined whether the mitochondrial effects of MOTS-c were associated with changes in mitochondrial fission-related proteins and mitochondrial maintenance pathways. MOTS-c did not significantly alter p-DRP1 Ser637, p-DRP1 Ser616, MID51, or MID49, although modest upward trends were observed for several markers (Fig. 4A and B). These findings suggest that the mitochondrial effects of MOTS-c are unlikely to be primarily driven by a strong shift in canonical DRP1-associated fission signaling. In contrast, MOTS-c significantly increased NRF2, phosphorylated AIFM1, RHOT1, and KIF5B (Fig. 4C and D). Because NRF2 is associated with stress-adaptive responses and RHOT1/KIF5B are involved in mitochondrial trafficking and intracellular transport, these data suggest that MOTS-c is associated with a mitochondrial stress-adaptive and trafficking-associated protein response.

**Figure 4:**
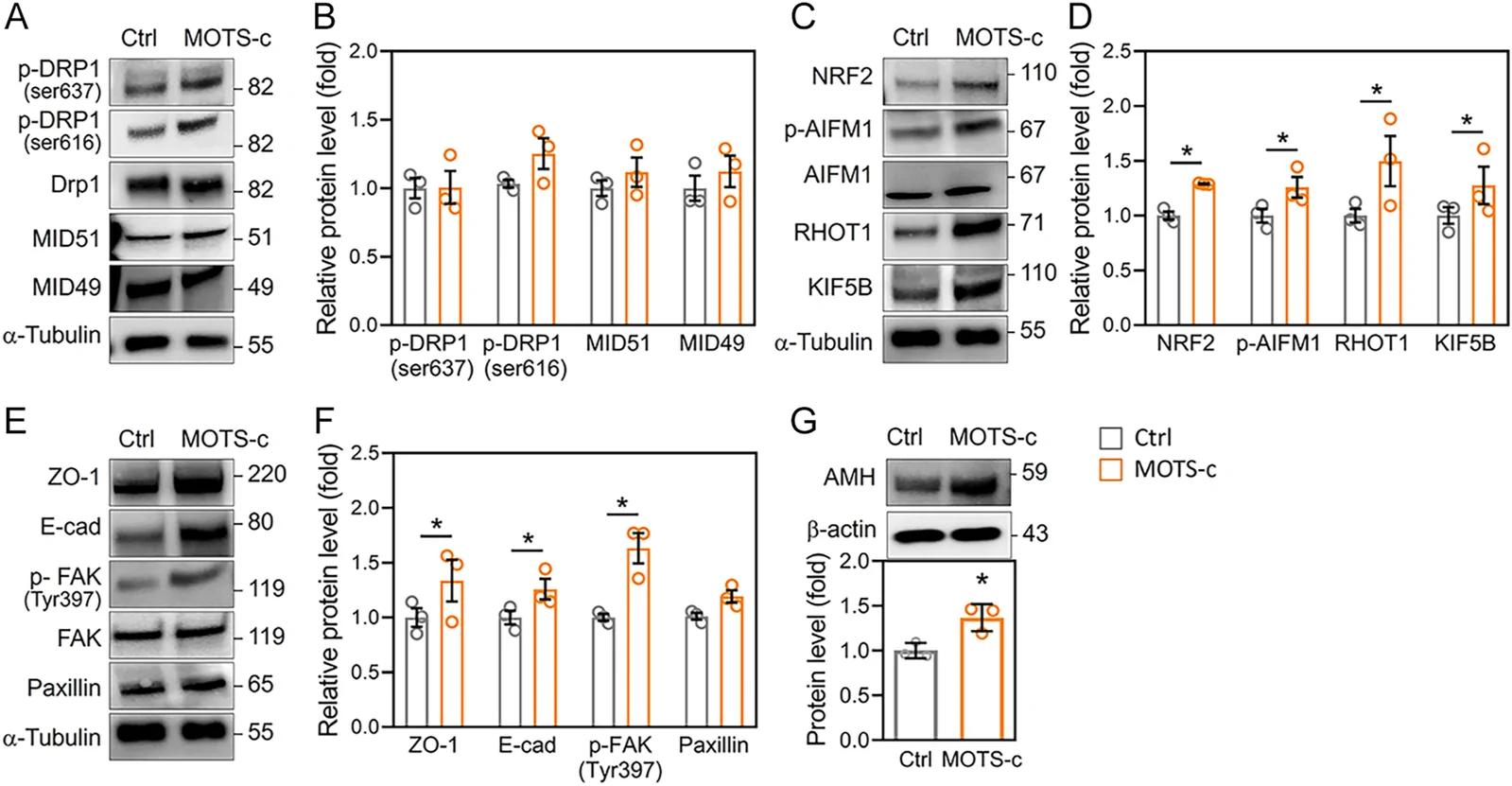
MOTS-c promotes mitochondrial protective/trafficking-associated proteins and granulosa support-associated markers. (A) Representative blots and (B) quantification of p-DRP1 Ser637, p-DRP1 Ser616, MID51, and MID49. MOTS-c did not significantly alter these fission-associated markers. (C) Representative blots and (D) quantification of mitochondrial protection/trafficking-associated proteins. MOTS-c significantly increased NRF2, the p-AIFM1/AIFM1 ratio, RHOT1, and KIF5B after treatment. (E) Representative blots and (F) quantification of adhesion- and junction-associated proteins. MOTS-c significantly increased ZO-1, E-cadherin, and the p-FAK Tyr397/total FAK ratio, whereas Paxillin showed an increasing trend without statistical significance. (G) Representative blot and quantification showing increased AMH protein expression after MOTS-c treatment. For phosphorylated proteins, p-AIFM1 and p-FAK Tyr397 were quantified relative to total AIFM1 and total FAK, respectively. Other protein levels were normalized to α-tubulin or β-actin as loading controls. *P < 0.05.

Because granulosa cell support of oocyte quality depends not only on mitochondrial function but also on cellular organization, we further examined junctional and adhesion-associated proteins. MOTS-c significantly increased ZO-1, E-cadherin, and phosphorylated FAK at Tyr397, whereas Paxillin showed an increasing tendency without reaching statistical significance (Fig. 4E and F). In addition, MOTS-c significantly increased AMH protein expression (Fig. 4G). These findings suggest that MOTS-c enhances selected components of granulosa cell junctional organization, adhesion signaling, and functional phenotype. Together with the mitochondrial and metabolic data, these results support the concept that MOTS-c improves both energetic and structural features of granulosa cell support function; because these experiments were performed in HGL5 cells alone, this granulosa-cell-intrinsic effect is reported independently of the oocyte-associated findings described below.

### MOTS-c partially ameliorates age-associated decline in oocyte maturation and mitochondrial function

To determine whether the mitochondrial-derived peptide MOTS-c could ameliorate the age-associated decline in oocyte quality, cumulus–oocyte complexes were collected from young (<12 weeks) and aged (>34 weeks) female mice. Following enzymatic removal of the surrounding cumulus cells, denuded oocytes from aged mice were randomly assigned to vehicle or MOTS-c treatment, while denuded oocytes from young mice served as a young reference group; all oocytes were maintained in *ex vivo* culture under matched conditions (Fig. 5A). Morphological examination identified oocytes retaining a compact granulosa cell layer that resisted enzymatic denudation (asterisks in Fig. 5B). Such residual granulosa-cell coverage was treated as a morphological observation rather than direct evidence of germinal vesicle-stage arrest. In the post-culture assessment, the proportion of oocytes exhibiting a first polar body was significantly lower in aged control oocytes than in young oocytes, and MOTS-c treatment was associated with a significantly higher post-culture PB1-positive proportion in aged oocytes relative to aged controls, although this proportion remained below that observed in young oocytes (Fig. 5C). Because oxidative stress and mitochondrial dysfunction are major contributors to the deterioration of oocyte quality during reproductive aging, intracellular ROS and mitochondrial activity-associated signal were assessed using DCFDA and CMXRos staining, respectively (Fig. 5D). Aged control oocytes showed markedly increased DCFDA fluorescence and decreased CMXRos fluorescence relative to young oocytes; MOTS-c treatment of aged oocytes decreased DCFDA fluorescence and increased CMXRos fluorescence toward young-like levels (Fig. 5E and F). Collectively, these results indicate that aged oocytes had a lower post-culture PB1-positive proportion, increased oxidative stress, and reduced mitochondrial activity-associated signal relative to young oocytes, and that *ex vivo* MOTS-c treatment improved these post-culture maturation-associated and mitochondrial/redox readouts without fully restoring young-reference values.

**Figure 5:**
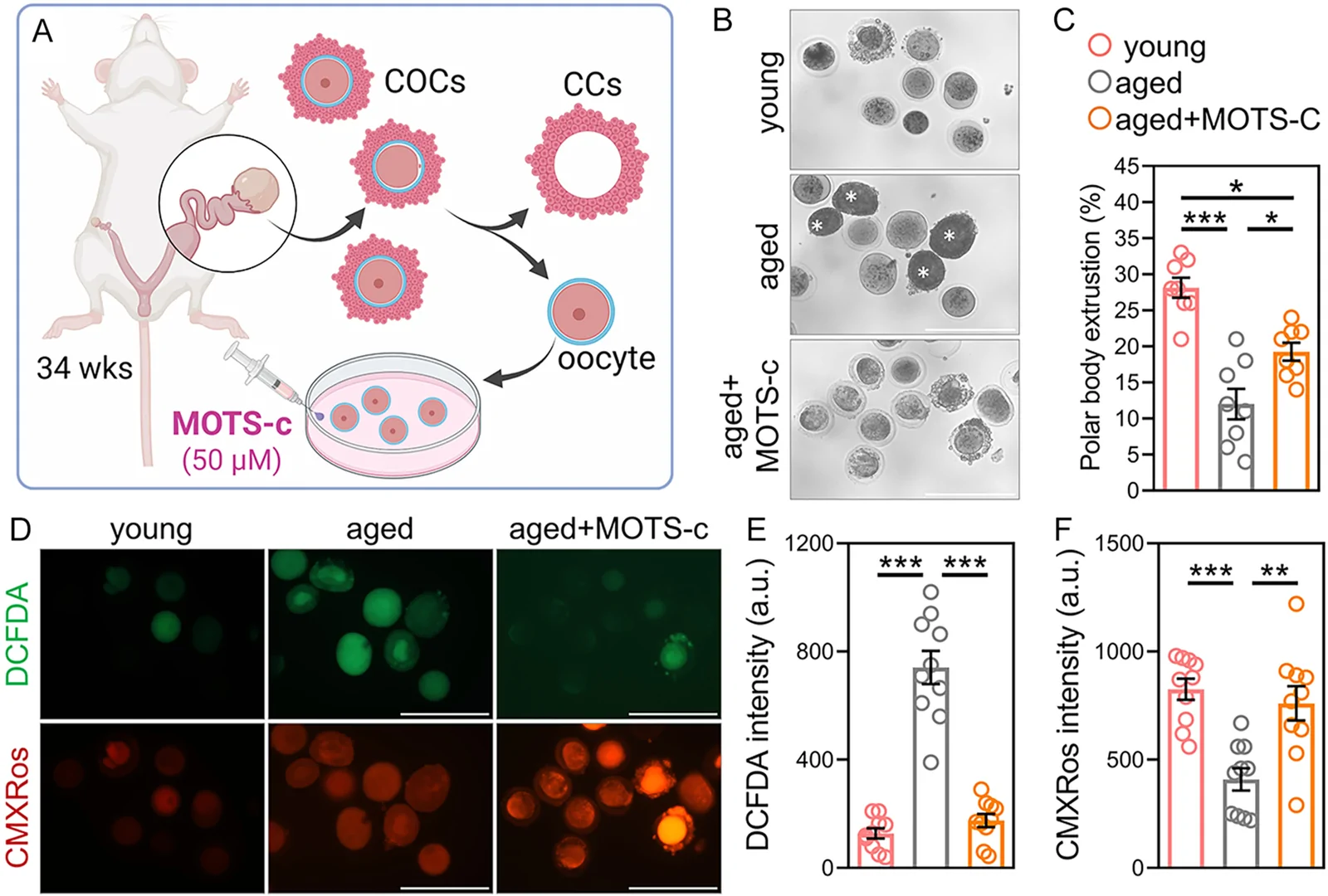
MOTS-c partially reverses the age-associated decline in oocyte maturation and mitochondrial activity in an *ex vivo* model. (A) Schematic illustration of the *ex vivo* oocyte experimental design. Cumulus–oocyte complexes were collected from young and aged mice; denuded oocytes from aged mice were treated with vehicle or MOTS-c during *ex vivo* culture, and denuded oocytes from young mice served as a young reference group. (B) Representative bright-field images of oocytes from young, aged, and aged + MOTS-c groups. Asterisks (*) indicate oocytes with a compact granulosa cell layer that resisted enzymatic denudation; this morphology alone does not establish meiotic stage. (C) Quantification of the first polar body extrusion rate across young, aged, and aged + MOTS-c groups. (D) Representative fluorescence images of oocytes stained with DCFDA and CMXRos across the three groups. Quantification of DCFDA (E) and CMXRos (F) fluorescence intensity (a.u.). Statistical comparisons were performed using one-way ANOVA with Tukey’s post hoc test. *P < 0.05, **P < 0.01, ***P < 0.001. Scale bar = 100 μm. For panels (E) and (F), fluorescence attributable to any residual granulosa cells was excluded from oocyte fluorescence-intensity quantification.

### Single-cell transcriptomics uncovers MOTS-c metabolic downregulation during primate ovarian aging

To examine in greater detail the transcriptomic basis for the age-associated metabolic trend introduced in Fig. 3, we analyzed a public single-cell RNA-seq dataset from young and aged cynomolgus monkey ovaries (GSE130664). Young and aged granulosa cells partially overlapped in UMAP space, indicating that aging was associated with a transcriptional state shift rather than complete separation of granulosa cell populations (Fig. 6A). Projection of the MOTS-c metabolic program score onto the UMAP showed heterogeneous but broadly distributed signal across granulosa cells (Fig. 6B). Cell-level comparison showed a higher distribution of the MOTS-c metabolic program score in young granulosa cells than in aged granulosa cells, although substantial overlap between the two groups was observed (Fig. 6C). Donor-level analysis showed a trend toward higher median MOTS-c metabolic program scores in young donors than in aged donors, with near-significant statistical support (Fig. 6D). Module-level decomposition indicated that this age-associated pattern was most consistently reflected by the metabolic program, whereas OXPHOS, mitochondrial protection/trafficking, and junction/support modules showed greater donor-level heterogeneity; the same modules also varied across inferred folliculogenesis stages (Fig. 6E). Granulosa-enriched cells used for these analyses were identified from the clustered dataset using canonical lineage markers, with the granulosa cluster showing enrichment of AMH, INHA, INHBA, AMHR2, CYP19A1, and FSHR relative to oocyte, stromal, endothelial, immune, and theca markers (Fig. 6F, G, H, I). These findings indicate that this nonhuman primate transcriptomic dataset provides supportive, but not definitive, evidence that the metabolic component of the MOTS-c-associated granulosa program is relatively enriched in young granulosa cells and may be attenuated with aging, although extrapolation from a nonhuman primate model to human ovarian aging warrants caution.

**Figure 6:**
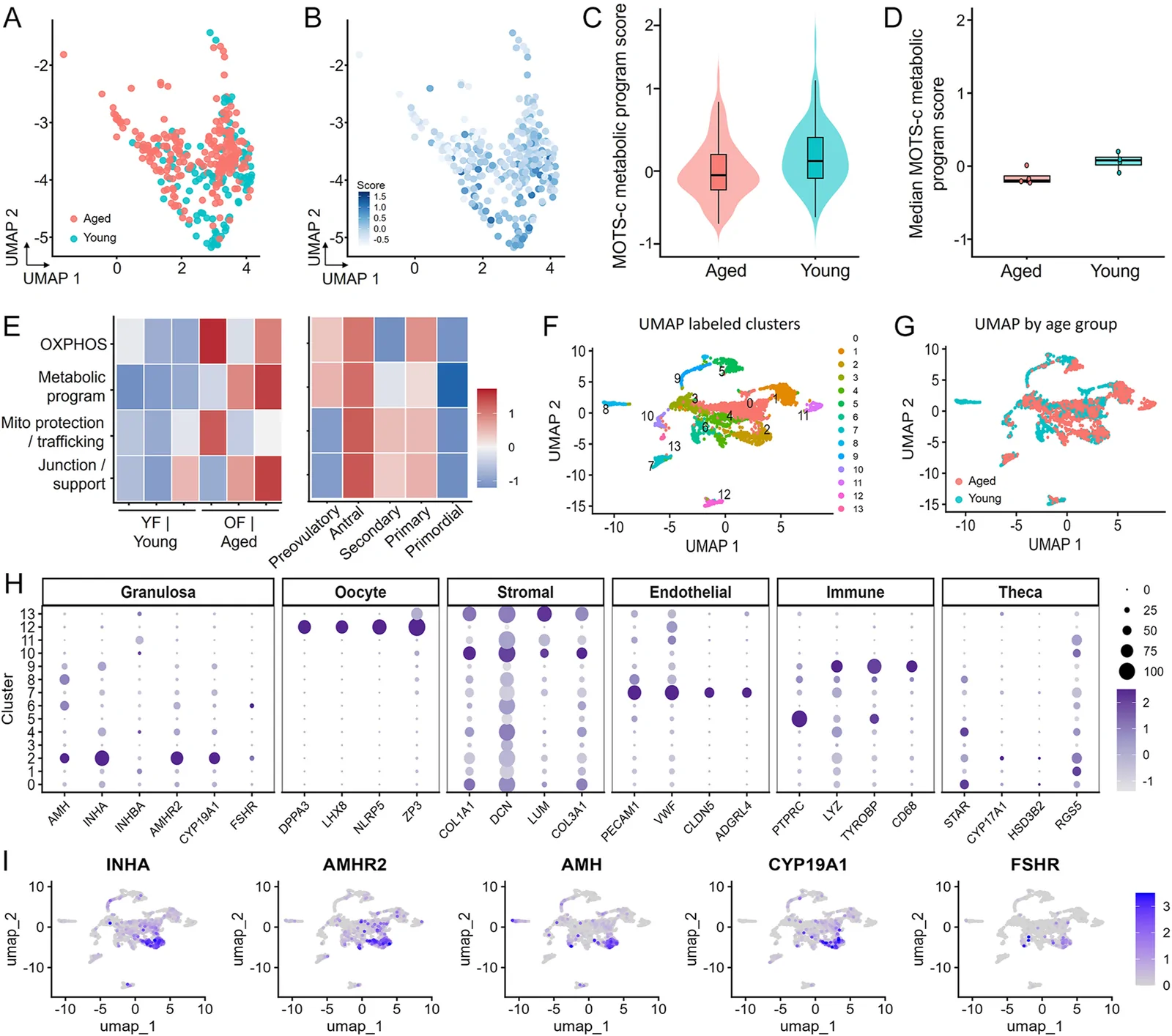
Public transcriptomic analysis supports a young-enriched trend in the MOTS-c metabolic program in granulosa cells. (A) UMAP visualization of granulosa cells from young and aged donors in GSE130664. Young and aged cells showed partial overlap, consistent with an age-associated state shift rather than complete cell separation. (B) UMAP projection of the MOTS-c metabolic program score. (C) Cell-level distribution of the MOTS-c metabolic program score in aged and young granulosa cells. (D) Donor-level comparison of median MOTS-c metabolic program scores. Each point represents one donor. Young donors showed a trend toward higher scores than aged donors. (E) Heatmaps showing relative enrichment of OXPHOS, metabolic, mitochondrial protection/trafficking, and junction/support modules across donors (left, young versus aged) and across inferred folliculogenesis stages (right). Scores were z-transformed within each module. The metabolic program showed the clearest young-enriched pattern, whereas other modules displayed greater heterogeneity. (F) UMAP of all cells colored by cluster identity and (G) by age group. (H) Dot plot of curated lineage markers across clusters; the granulosa cluster was enriched for AMH, INHA, INHBA, AMHR2, CYP19A1, and FSHR relative to oocyte, stromal, endothelial, immune, and theca markers. (I) Feature plots of representative granulosa markers (INHA, AMHR2, AMH, CYP19A1, FSHR).

## Discussion

Ovarian aging is closely associated with reduced granulosa cell metabolic support, mitochondrial dysfunction, oxidative stress, and reduced oocyte quality. In this study, we show that MOTS-c improves several aging-associated features of granulosa cell dysfunction, including mitochondrial mass, respiratory reserve capacity, metabolic enzyme expression, mitochondrial protective/trafficking-associated proteins, and junctional and adhesion-associated markers. In a separate line of evidence, using denuded oocytes from young and aged mice, we found that *ex vivo* MOTS-c treatment partially improved oocyte-associated outcomes relative to aged controls, including increased polar body extrusion, reduced DCFDA intensity, and increased CMXRos fluorescence, although these outcomes did not fully reach young-oocyte levels. Because MOTS-c was applied directly to denuded oocytes without accompanying granulosa or cumulus cells, these oocyte-associated effects and the granulosa cell findings described above are best interpreted as independent, parallel benefits of MOTS-c rather than evidence of a coordinated granulosa–oocyte axis. Together, these findings suggest that MOTS-c does not act only as an antioxidant-like molecule but instead promotes a metabolic, mitochondrial, and structural support program in aging-associated granulosa cells, together with direct protective effects on aged oocytes.

Previous work in human mural granulosa cells showed that reproductive aging is associated with reduced mitochondrial membrane potential, impaired oxidative phosphorylation capacity, and abnormal mitochondrial features, supporting mitochondrial deterioration as a relevant component of granulosa cell aging (Liu *et al.* 2017). Mitochondrial and metabolic dysfunction in cumulus and granulosa cells has also been linked to reduced reproductive capacity and oocyte competence in aging-related contexts (Lu *et al*. 2022, Morimoto *et al.* 2024). Our findings extend these observations by showing that MOTS-c improves respiratory reserve and ATP-coupled respiration in an aging-associated granulosa cell model while maintaining low ROS-related fluorescence. Importantly, the increase in mitochondrial mass-associated fluorescence occurred alongside higher ATP-linked respiration and spare respiratory capacity and lower proton-leak respiration. This combination is compatible with coordinated improvement in mitochondrial bioenergetic performance rather than an increase in mitochondrial content alone. Selective removal of dysfunctional mitochondria through mitochondrial quality-control pathways, including mitophagy, is one possible explanation. However, mitochondrial turnover, mitophagic flux, and clearance of damaged mitochondria were not directly measured; therefore, these findings cannot establish that MOTS-c induces mitophagy. This profile is also consistent with evidence that oocytes preserve mitochondrial activity while minimizing ROS generation (Rodríguez-Nuevo *et al.* 2022). In this context, MOTS-c fits within the broader role of mitochondrial-derived peptides in cellular energetic adaptation. MOTS-c has been shown to translocate to the nucleus under metabolic stress and regulate nuclear gene expression through AMPK-dependent mechanisms (Kim *et al.* 2018*a*). Mitochondrial-derived peptides, including MOTS-c, also modulate mitochondrial respiration in senescent-cell models (Kim *et al.* 2018*b*), and MOTS-c has been reported to restore mitochondrial respiration in a type 2 diabetic heart model, although ROS-related responses may differ across tissues and disease contexts (Pham *et al.* 2025).

The metabolic enzyme data suggest that MOTS-c induces substrate-handling changes that may support the improved mitochondrial respiratory phenotype. MOTS-c increased HK2, ENO1, and PKM1, indicating increased glycolytic processing capacity. This response did not represent a simple shift toward lactate-producing glycolysis, as LDHA was reduced whereas LDHB was increased. Because LDHA is generally associated with pyruvate-to-lactate conversion and LDHB can support lactate-to-pyruvate oxidation in oxidative metabolic contexts, this LDH isoform pattern suggests altered lactate–pyruvate handling rather than uniform activation of anaerobic glycolysis. In parallel, increased PDHA, CS, ACO1, and IDH1 suggest greater readiness for pyruvate utilization and selected TCA-cycle-associated metabolism. This interpretation is consistent with experimental evidence that follicular somatic cell metabolism is required for oocyte maturation and developmental competence (Li *et al.* 2011). Cumulus-cell glucose metabolism supports oocyte maturation by generating pyruvate, NADPH, and other metabolites derived from glycolysis or the pentose phosphate pathway, and lactate metabolism in cumulus cells can contribute to maturation through LDH-catalyzed oxidation to pyruvate (Xie *et al.* 2016, Wen *et al.* 2020). Granulosa cell metabolism at ovulation was also shown to correlate with oocyte competence and to be disrupted by aging and obesity (Morimoto *et al.* 2024). Thus, the MOTS-c-induced increase in glycolytic, lactate-handling, and pyruvate–TCA-associated proteins may represent a metabolic support signature in aging-associated granulosa cells. Future isotope-tracing studies will be needed to determine whether this protein-level remodeling corresponds to altered carbon flux into mitochondrial oxidation.

Another finding is that MOTS-c did not strongly alter canonical mitochondrial fission-associated markers, including p-DRP1 Ser637, p-DRP1 Ser616, MID51, and MID49. MiD49 and MiD51 have been reported to recruit DRP1 and participate in DRP1-dependent mitochondrial fission (Losón *et al.* 2013, Otera *et al.* 2016) , suggesting that MOTS-c does not mainly act through broad activation of the canonical fission machinery. Instead, MOTS-c increased NRF2, phosphorylated AIFM1, RHOT1, and KIF5B, indicating a shift toward mitochondrial stress-adaptive and trafficking-associated protein responses. RHOT1/Miro1 and KIF5B are components of microtubule-based mitochondrial transport machinery mediated in part by Miro/TRAK adapter complexes (Baltrusaitis *et al.* 2023, Canty *et al.* 2023). The increase in RHOT1 and KIF5B may therefore indicate greater mitochondrial distribution capacity in MOTS-c-treated granulosa cells. Because mitochondrial movement, subcellular localization, and transzonal projection-associated mitochondrial positioning were not directly measured, this interpretation should be viewed as a trafficking-associated protein signature rather than direct evidence of enhanced mitochondrial transport.

The increase in junctional and adhesion-associated proteins adds functional relevance to these findings. MOTS-c increased ZO-1, E-cadherin, and phosphorylated FAK Tyr397, while Paxillin showed an increasing trend. Adhesion- and junction-related proteins have been characterized in ovarian follicles, with adherens junction-associated structures contributing to granulosa cell–granulosa cell and granulosa cell–oocyte organization (Mora *et al.* 2012). E-cadherin has been implicated in ovarian developmental events and primordial follicle maintenance in mouse models (Yan *et al.* 2019), and ZO-1/ZO-2 expression has been linked to porcine oocyte maturation and early embryonic development (Cao *et al.* 2021). FAK activation has also been reported in ovarian follicular granulosa cells and cumulus–oocyte complexes, where it participates in cumulus expansion and ovulation-associated processes (Sakurai *et al.* 2012). Therefore, the MOTS-c-induced increase in selected adhesion- and junction-associated proteins may reflect reinforcement of the somatic structural environment required for oocyte support. These findings should not be interpreted as complete restoration of follicular architecture because our experiments were conducted in an *in vitro* granulosa cell model. Rather, they indicate that MOTS-c improves selected structural and adhesion-related components relevant to granulosa cell support function.

Consistent with the concept that metabolic and mitochondrial interventions can influence reproductive aging outcomes, *ex vivo* MOTS-c treatment partially improved oocyte-associated outcomes relative to aged controls, increasing polar body extrusion, reducing DCFDA fluorescence, and increasing CMXRos intensity, although these outcomes did not fully reach young-oocyte levels. Because first polar body status was assessed after the 24-h *ex vivo* period, the percentage of PB1-positive oocytes should be interpreted as an endpoint maturation-associated measure rather than proof that each PB1-positive oocyte newly completed meiosis during MOTS-c exposure. For example, NAD+ repletion by nicotinamide mononucleotide supplementation was shown to improve oocyte quality, embryo development, and fertility-related outcomes, including cumulative live birth rate, in aged mice (Bertoldo *et al.* 2020). CoQ10 supplementation has also been reported to restore oocyte mitochondrial function and fertility, including functional fertilization and litter outcomes, during reproductive aging in mice (Ben-Meir *et al.* 2015). Because these studies evaluated functional fertility endpoints, including fertilization rates, blastocyst formation, and live birth or litter outcomes, that were not assessed in the present study, our findings should be considered complementary rather than directly comparable in magnitude; whether MOTS-c can similarly restore functional fertility remains untested and will require future *in vivo* studies. In the context of mitochondrial-derived peptides, MOTS-c has been reported to reduce prepubertal cyclophosphamide-induced reproductive injury in male mice, supporting the relevance of MDPs in reproductive stress models (Wang *et al.* 2024). Our study extends this concept to an aging-associated female reproductive setting by identifying MOTS-c-induced improvements in granulosa cell bioenergetics and structural support markers and, independently, protective effects of MOTS-c on oocyte-associated functional readouts. Because the *ex vivo* oocyte experiment does not fully recapitulate the intact follicular microenvironment, future *in vivo* studies are needed to determine whether MOTS-c can improve fertility outcomes, follicular development, embryo competence, and offspring-related endpoints in aged females.

The nonhuman primate transcriptomic analysis provides supportive cross-species context for the experimental findings. Reanalysis of GSE130664 (young and aged cynomolgus monkey ovaries) showed that the experimentally defined MOTS-c metabolic program was relatively enriched in young granulosa cells, with donor-level analysis showing a young-enriched trend (Wang *et al.* 2020). Module-level decomposition indicated that this age-associated pattern was mainly driven by the metabolic program, whereas OXPHOS, mitochondrial protection/trafficking, and junction/support modules showed greater donor-level heterogeneity (Fig. 6E). These same programs also varied across inferred folliculogenesis stages, indicating that they are physiologically engaged during normal follicle development (Fig. 3A). Because these public datasets do not include MOTS-c treatment, the transcriptomic analysis should be interpreted as external support for the metabolic axis rather than independent validation of the complete mechanistic model.

Based on these findings, we propose a model in which MOTS-c improves aging-associated granulosa cell dysfunction through metabolic, mitochondrial, and structural remodeling (Fig. 7). In this model, MOTS-c enhances mitochondrial energetic fitness and respiratory reserve without increasing ROS-associated fluorescence, promotes protein-level remodeling of the glycolysis–lactate–pyruvate–TCA axis, increases mitochondrial stress-adaptive and trafficking-associated protein responses, and strengthens selected junctional and adhesion-associated markers. These changes may improve the somatic support environment required for oocyte maturation, as supported by improved polar body extrusion and mitochondrial/redox-associated readouts in aged *ex vivo* oocytes.

**Figure 7:**
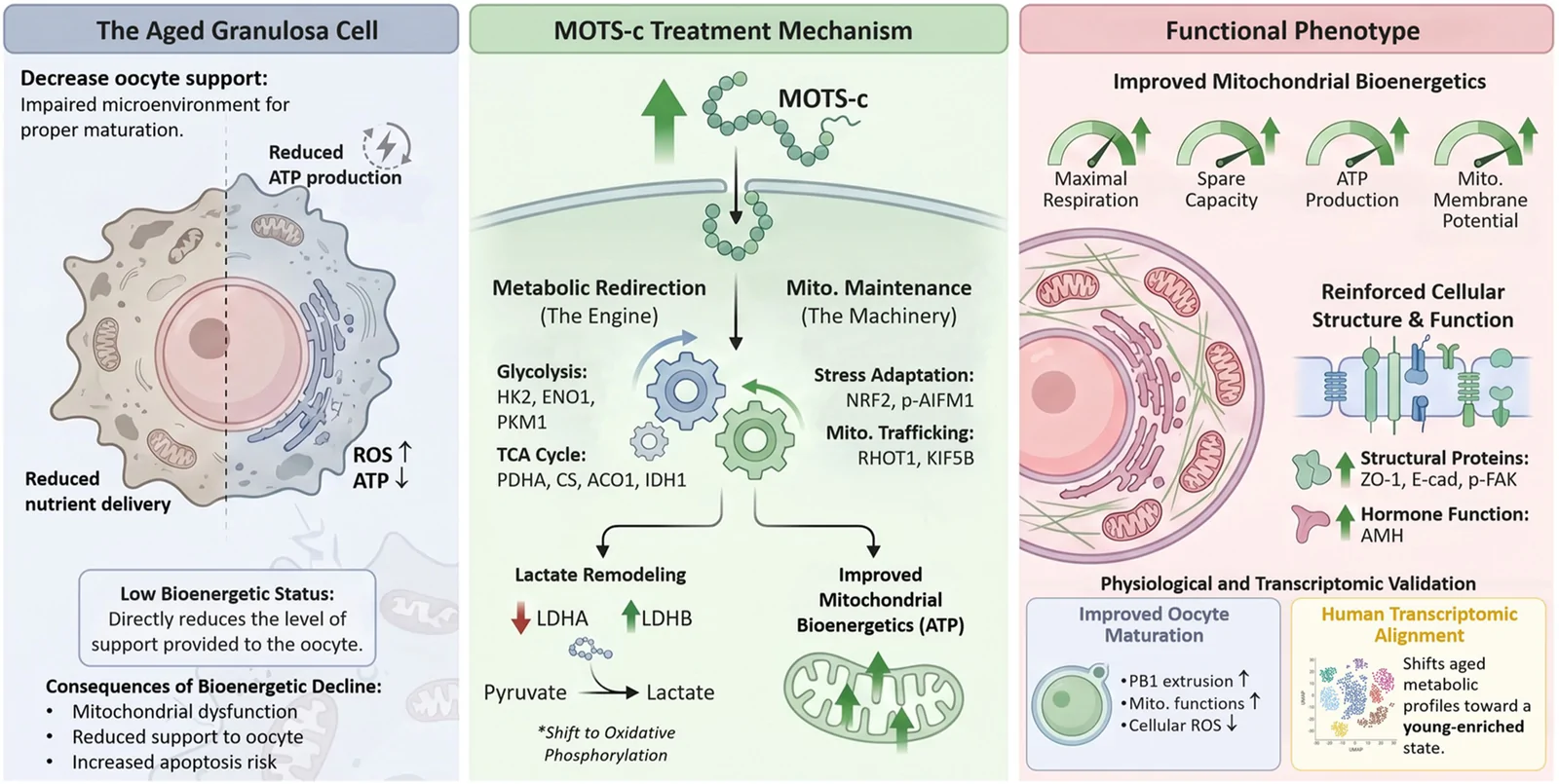
Proposed model of MOTS-c-mediated metabolic–mitochondrial–structural remodeling in aging-associated granulosa cells. MOTS-c promotes coordinated remodeling of granulosa cell metabolic support, mitochondrial maintenance, and structural organization. This includes protein-level remodeling of glycolytic/lactate and pyruvate–TCA-associated pathways, increased mitochondrial stress-adaptive and trafficking-associated proteins, and enhanced junctional/adhesion-associated markers. These changes may contribute to improved mitochondrial respiratory reserve and oocyte maturation-associated outcomes during reproductive aging.

Several limitations should be noted. Late-passage HGL5 cells provide a practical aging-associated granulosa cell model but do not fully recapitulate primary granulosa cells from aged ovaries; senescence in this model was supported by increased SA-β-galactosidase activity and p16/p27 expression relative to early-passage cells (Supplementary Fig. S1A and B), although additional markers such as γH2AX were not assessed. Our metabolic interpretation is based on protein abundance and respiratory profiling; isotope tracing or substrate-dependency assays will be required to determine whether MOTS-c directly redirects glucose- or lactate-derived carbon into pyruvate oxidation and the TCA cycle. Increased mitochondrial mass-associated fluorescence does not establish biogenesis or the selective clearance of dysfunctional mitochondria; neither mitophagy nor mitochondrial turnover was directly quantified. Although MOTS-c increased RHOT1 and KIF5B, mitochondrial trafficking was not directly assessed by live-cell imaging or subcellular localization analysis. AMH was used as a granulosa functional marker and should not be interpreted as evidence of restored ovarian reserve. The proposed involvement of AMPK-, PGC-1α-, or NRF2-associated signaling in mediating MOTS-c’s mitochondrial and metabolic effects was not directly tested using pharmacological inhibitors, siRNA, or genetic loss-of-function approaches; these mechanistic interpretations should therefore be regarded as associative rather than causally established, pending future loss-of-function studies. The *ex vivo* oocyte experiments evaluated meiotic maturation, redox, and mitochondrial activity-associated readouts only; whether MOTS-c improves functional fertility endpoints such as fertilization, blastocyst formation, or live birth remains untested. Formal normality testing was not performed before parametric statistical comparisons, which is noted as a methodological limitation. The nonhuman primate single-cell transcriptomic analysis was exploratory, with limited donor-level statistical power, and cross-species extrapolation to human ovarian aging warrants caution; therefore, the young-enriched MOTS-c metabolic program should be interpreted as supportive rather than definitive validation. Although residual granulosa cell fluorescence was excluded from oocyte fluorescence quantification, the possibility that persistent surrounding granulosa cells affected oocyte physiology cannot be fully ruled out.

## Conclusion

In conclusion, MOTS-c improves aging-associated granulosa cell dysfunction by enhancing mitochondrial mass, respiratory reserve, metabolic remodeling, mitochondrial protective and trafficking-associated proteins, and selected structural support markers. In an independent line of evidence, *ex vivo* MOTS-c treatment of denuded oocytes from aged mice partially improved meiotic maturation-related outcomes relative to aged controls, without fully restoring young-oocyte levels. Rather than acting only as an antioxidant, MOTS-c appears to promote a metabolic, mitochondrial, and structural support program in granulosa cells, together with direct protective effects on aged oocytes, providing a basis for future studies of mitochondria-derived peptide strategies to improve oocyte quality during reproductive aging.

## Supplementary materials



## Declaration of interest

The authors declare that the research was conducted in the absence of any commercial or financial relationships that could be construed as a potential conflict of interest.

## Funding

This research was funded by the National Science and Technology Council (114-2628-B-075B-001-MY3 and 114-2314-B-075B-004-MY3), Kaohsiung Veterans General Hospital (KSVGH-115-052, 056, 063, 069), and the Health Taiwan Sprout Project, Ministry of Health and Welfare (HTS-114-115-A1-0016).

## Author contribution statement

C-JL and S-HC conceived the study and drafted the manuscript. S-HC and L-TL performed the experiments and analyzed the data. P-HL and BW contributed to data collection and interpretation. Z-HW and K-HT supervised the study and provided critical revisions. All authors contributed to the discussion of the results and approved the final version of the manuscript.

## Ethics declaration

All animal experiments and procedures performed in this study were approved by the Institutional Animal Care and Use Committee of Kaohsiung Veterans General Hospital (Approval No. IACUC-2508-2807-25002). Ethical approval and consent to participate were not applicable to the human cell line (HGL5) and public transcriptomic datasets used in this study because no human clinical subjects were directly involved.

## Data availability

The experimental datasets generated and analyzed during the current study are available from the corresponding author upon reasonable request. The public transcriptomic datasets analyzed in this study are openly available in the NCBI Gene Expression Omnibus (GEO) repository under accession numbers GSE130664 and GSE107746.
