# Supplementary material for: The mitochondrial-derived peptide MOTS-c improves bioenergetics in aging-associated granulosa cells and oocytes
Source: Reprod Fertil. 2026 Oct 27;7(4):RAF260161. doi: 10.1530/RAF-26-0161 (PMC13726400; doi:10.1530/RAF-26-0161)
Supplement: Supplementary file 1 [file RAF-26-0161_supplementary_figure_1.pdf]

The mitochondrial-derived peptide MOTS-c Improves Bioenergetics to Ameliorate Ovarian Aging

Kuan-Hao Tsui [1, 2, 3, 8,9,*], Shih-Hsuan Cheng [1,†], Li-Te Lin [1,2,3,4,5], Pei-Hsuan Lin[1,2,3], Boyang Wang [6], Zhi-Hong Wen[7,8], and Chia-Jung Li [1,2,5,8,†]

1. Department of Obstetrics and Gynecology, Kaohsiung Veterans General Hospital, Kaohsiung 813, Taiwan;

2. Institute of Biopharmaceutical Sciences, National Sun Yat-sen University, Kaohsiung 804, Taiwan;

3. Department of Obstetrics and Gynecology, National Yang-Ming University School of Medicine, Taipei 112, Taiwan;

4. School of Medicine, College of Medicine, National Sun Yat-sen University, Kaohsiung 804, Taiwan;

5. Center of General Education, Cheng Shiu University, Kaohsiung 833, Taiwan.

6. Sundial Initiative, Palo Alto, CA, 94304, USA;

7. Department of Marine Biotechnology and Resources, National Sun Yat-sen University, Kaohsiung 804, Taiwan;

8. National Museum of Marine Biology & Aquarium, Pingtung 944, Taiwan;

*****Correspondence:

Chia-Jung Li.: cjli@vghks.gov.tw, and nigel6761@gmail.com

Department of Obstetrics and Gynecology, Kaohsiung Veterans General Hospital, Kaohsiung 813, Taiwan

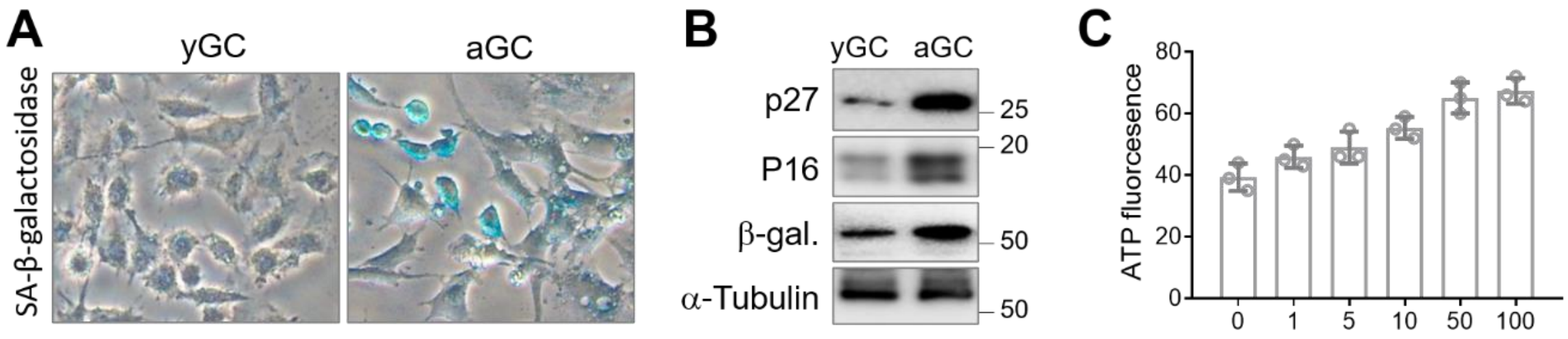

Supplementary figure 1. Validation of the late-passage HGL5 senescence model and MOTS-c dose-response.

(A) SA-β-galactosidase staining of early-passage (yGC) and late-passage (aGC) HGL5 cells, showing increased blue staining in aGC. (B) Representative immunoblots of p27, p16, and SA-β-galactosidase (β-gal.) in yGC and aGC, with α-tubulin as loading control, showing increased expression of senescence markers in aGC. (C) Dose-response curve of ATP-associated fluorescence in HGL5 cells treated with MOTS-c (0-100 μM, 24 h), showing a near-saturating response at 50 μM.

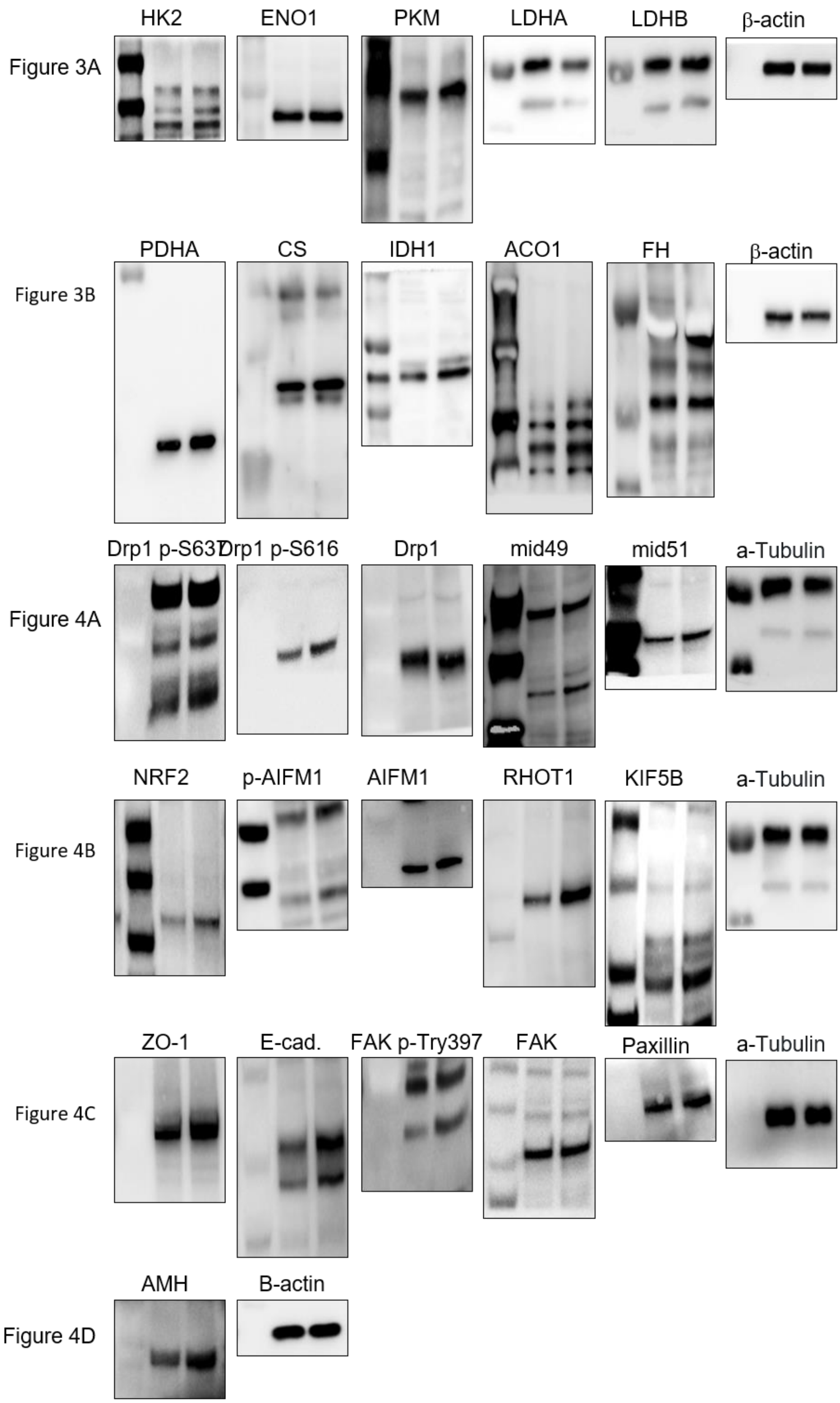

Supplementary Figure S1. uncropped blot
